# Integrating dynamic modeling and continuous cultivation to enhance recombinant β-glucosidase production in *Komagataella phaffii*

**DOI:** 10.1186/s40643-026-01147-9

**Published:** 2026-10-15

**Authors:** Eduardo Rocco-Martínez, Johan Quezada, Julio Berrios, Kenneth H. Wolfe, Alvaro Díaz-Barrera, Carlos Martínez, Stephanie Braun-Galleani

**Affiliations:** 1 Escuela de Ingeniería Bioquímica, Pontificia Universidad Católica de Valparaíso, Brasil 2085, 2362803 Valparaíso, Chile; 2 UCD Conway Institute and School of Medicine, University College Dublin, Dublin 4, Ireland; 3 Center for Interdisciplinary Research in Biomedicine, Biotechnology and Well-Being (CID3B), Pontificia Universidad Católica de Valparaíso, Brasil 2950, 2340025 Valparaíso, Chile

**Keywords:** *Pichia pastoris*, *Komagataella phaffii*, β-Glucosidase, Hypothermia, Chemostat

## Abstract

Cultivation under hypothermia has emerged as an interesting strategy for enhancing recombinant protein production in *Komagataella phaffii*, yet predictive approaches capable of integrating the effects of temperature and cell growth rate for rational bioprocess optimization remain limited. In this work, we investigated the influence of cultivation temperature on recombinant β-glucosidase (BGL1) production in the hybrid *K. phaffii* strain S467 by combining bioprocess characterization with dynamic mathematical modeling. Batch cultures performed at 20, 25 and 30 °C showed that hypothermia increased recombinant protein secretion while reducing biomass formation, revealing a trade-off between cell growth and product formation. To quantitatively describe these dynamics, a Droop-based model incorporating delayed coupling between substrate uptake and biomass synthesis was fitted using the experimental data. Model simulations predicted that continuous cultivation at 20 °C and a dilution rate of 0.045 h^−1^ maximized the total secreted protein volumetric productivity. Chemostat experiments at selected conditions yielded biomass concentrations and total secreted protein productivities close to the simulated values, supporting the model-predicted trends and its potential as a tool for process optimization. Interestingly, the highest specific BGL1 activity was observed at the highest dilution rate despite a lower *bgl1* transcript level and secreted protein production, suggesting that cultivation conditions influence recombinant protein traits through mechanisms beyond transcriptional regulation. Collectively, these results show that integrating experimental bioprocess characterization, predictive dynamic modeling and targeted molecular analyses provide an effective framework for rational optimization of recombinant protein production in this biotechnologically relevant yeast.

**Graphical abstract:**

**Figure:**
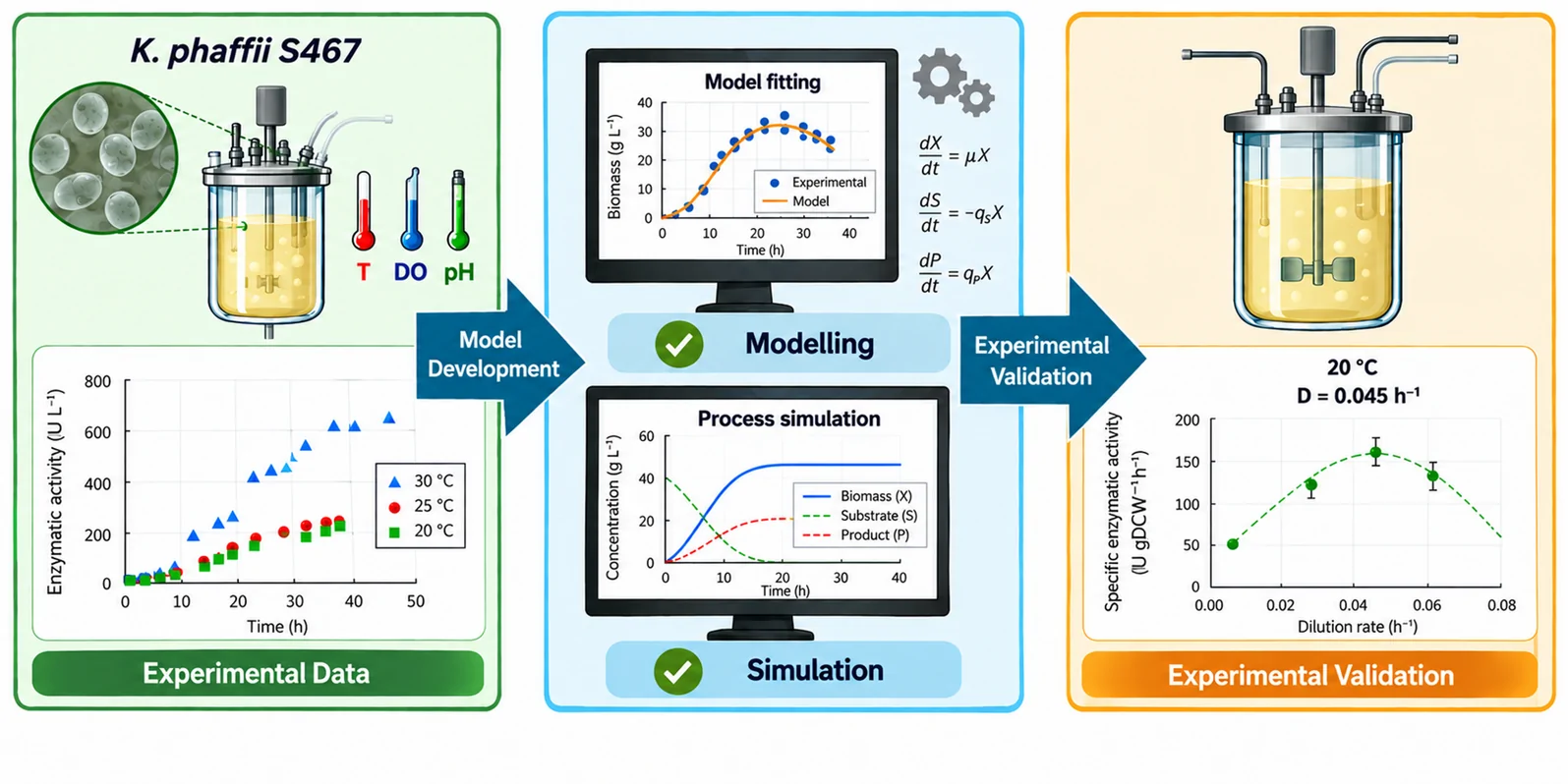

**Supplementary Information:**

The online version contains supplementary material available at https://doi.org/10.1186/s40643-026-01147-9.

## Introduction

*Komagataella phaffii* (previously known as *Pichia pastoris*) is currently one of the most widely used recombinant protein expression systems at both laboratory and industrial scale (Zhu et al. 2019; Gao et al. 2025). *K. phaffii* can grow rapidly to high cell densities in simple, inexpensive media, and it can perform post-translational modifications essential for protein functionality. Additionally, its secretory machinery can reach recombinant protein production levels of up to approximately 80% of the total secreted protein (García-Ortega et al. 2019; Kastberg et al. 2022). Moreover, its *Generally Recognized as Safe (GRAS)* status by the *Food and Drug Administration* and its *Qualified Presumption of Safety (QPS)* status by the *European Food Safety Authority*, addressing safety concerns for humans, animals and the environment (Spohner et al. 2015; Koutsoumanis et al. 2022) expand its usability for producing numerous recombinant proteins in a continuously increasing market (Khlebodarova et al. 2024). Recombinant protein production levels vary with the carbon source used, and higher titers of recombinant proteins, as well as biomass, are generally obtained when glucose is supplemented (Hou et al. 2022).

Given the importance of *K. phaffii* as a biotechnological tool, continuous advances for improving its bioprocess performance have been developed over the last two decades encompassing both cell engineering and optimization of expression and cultivation strategies (Patra et al. 2021; Bustos et al. 2022; Li et al. 2024; Lv and Cai 2025). In addition, an often-overlooked aspect that can play a relevant role is the development and use of novel strains, with distinctive genetic traits. This feature has been scarcely studied for this species, counting with very few genetically divergent strains that can be further improved for biotechnological applications (Braun-Galleani et al. 2019). The strain used in this study (S467) was generated by a genetic cross between two parental strains (the laboratory strain CBS7435 and the wild isolate strain NRRL Y-17741), both expressing β-glucosidase (*bgl1*) under the control of *pGAP*. S467, one of the segregants derived from this cross, harbors the *IRA1* gene variant from the wild isolate strain, an allele that has been associated with enhanced β-glucosidase production (Offei et al. 2022). Recent work from our group showed that S467 has improved BGL1 specific productivity in comparison to an equivalent strain generated in the CBS7435 background, highlighting the potential of this novel hybrid strain as a new host (Velastegui et al. 2025).

In this context, understanding growth kinetics in novel strains is essential for elucidating their behavior and potential for different bioprocess applications. Kinetic models enable the representation of this behavior by considering physiological and metabolic aspects of the microorganism (Montesinos et al. 1995; Nielsen 1996). The most used model (Monod) describes the relationship between the specific growth rate and the concentration of the limiting substrate in the medium. When high substrate concentrations may lead to inhibitory effects, the Haldane model can be applied, which has been particularly useful for *K. phaffii* cultivation using substrates like methanol (Barrigon et al. 2015; Canales et al. 2018; Ergün et al. 2021). However, a constraint observed with these models is the assumption that the specific growth rate depends directly on the concentration of substrate present in the medium (Barrigon et al. 2015). In contrast, the model proposed by Droop introduces the concept of cell quota, defined as the amount of intracellularly accumulated substrate per unit of biomass, as a means of accounting for cell growth that does not depend directly on the substrate available in the medium, but rather on the amount of nutrients previously stored within the cell (Droop 1968). Although originally developed for microalgae, the Droop model has also been previously applied to yeasts (Robles-Rodríguez et al. 2018).

Systems such as continuous cultures, where cells can be maintained under non-transient conditions (steady state), have become valuable tools for bioprocess characterization and subsequent optimization (Nieto-Taype et al. 2020). Among the different cultivation parameters, temperature is a highly relevant variable during the standardization of recombinant protein production processes (Berrios et al. 2017), as it affects several important cellular processes, including carbon metabolism, stress response, and protein folding. The most common temperature condition used for *K. phaffii* cultivation is 30 °C. Nevertheless, Dragosits *et al*. evaluated the effect of temperature on *K. phaffii* X-33 expressing an antibody Fab fragment using single-stage chemostat cultures, finding that a decrease in temperature from 30 °C to 20 °C resulted in a threefold increase in specific protein productivity (q_P_) (Dragosits et al. 2009). Similarly, a separate study reported a higher yield of recombinant human interleukin-10 using extended methanol-induction times in fed-batch cultivation at 20 °C, in comparison to 30 °C (Zhong et al. 2014). Cultivation at lower temperatures has also been reported to reduce proteolytic degradation, alleviate endoplasmic reticulum (ER) stress and improve protein folding (Li et al. 2001; Jahic et al. 2003; Zhong et al. 2014). Berrios *et al*. evaluated the effect of temperature in continuous cultures using a co-substrate strategy with methanol as the inducer for *Rhizopus oryzae* lipase production in *K. phaffii* X-33, observing that a temperature decrease from 30 °C to 22 °C did not improve either specific (q_P_) or volumetric (Q_P_) productivity; however, it enhanced biomass concentration compared to the higher temperature (Berrios et al. 2017). A similar effect on biomass accumulation was observed by Joseph *et al*. when growing *K. phaffii* GS115 expressing thaumatin II in shake flasks using two common media (FM22 and BMGY) (Joseph et al. 2022). In this study, the authors also reported lower recombinant protein secretion levels at 20 °C. Altogether, these results indicate that the effect of temperature depends on both the recombinant protein expressed and the cultivation strategy applied.

The aim of this work was to investigate the effect of cultivation temperature on cell growth and recombinant protein production in the novel hybrid strain S467, to identify conditions that maximize secreted protein specific productivity within the experimental range examined. A mathematical model was fitted with experimental data from batch cultures to support process optimization by reducing the experimental design space and focusing the experimental evaluation in chemostats on the most relevant conditions.

## Methods

### Strains

*K. phaffii* strain S467 was used in this study. This strain was developed and reported previously (Offei et al. 2022) and it expresses the *bgl1* gene from *Thermoascus aurantiacus* (GenBank accession number DQ114397.1), expressing the secreted β-glucosidase BGL1. The S467 genome sequence assembly is available in NCBI (accession number ASM2301493v1). A standardized working bank for bioreactor cultivations was generated and stored in glycerol stocks at − 80 °C.

### Flask cultivation

The inoculum for bioreactor cultivations was grown from a cryostock vial, which was used to inoculate a 500 mL Erlenmeyer flask containing 100 mL of YNB-G medium (per liter: 1.7 g yeast nitrogen base without amino acids (Becton, Dickinson and Company, Sparks, MD, USA), 0.34 g K_2_HPO_4_, 4.22 g KH_2_PO_4_, 5 g (NH_4_)_2_SO_4_ and 15 g glucose). This culture was incubated at 30 °C in an orbital shaker (LabTech LSI-3016 A) at 200 rpm for 24 h until reaching an OD_600_ of ~ 1.

### Batch cultivation

Subsequently, batch cultivations were carried out in duplicate in a 2 L Applikon My-control bioreactor fitted with pH, dissolved oxygen tension (DOT) and temperature control, and MFC for gas mix control (Getinge AB, Gothenburg, Sweden) with a working volume of 1.5 L. FM22 medium (per liter: 5 g (NH_4_)_2_SO_4_, 42.9 g K_2_HPO_4_, 14.3 g K_2_SO_4_, 11.7 g MgSO_4_·7H_2_O, 1 g CaSO_4_·2H_2_O, 1 mL PTM4 trace elements solution and 1 mL of 0.2 g L^−1^ stock solution of biotin (Sigma-Aldrich, St. Louis, MO, USA). This medium was supplemented with 40 g L^−1^ glucose. The glucose and salts were sterilized separately, while the PTM4 and biotin solutions were sterilized by filtration and added aseptically to the cooled medium. The PTM4 solution was prepared with the following composition (per liter): 2 g of CuSO_4_·5H_2_O, 0.08 g of NaI, 3 g of MnSO_4_·H_2_O, 0.2 g of Na_2_MoO_4_·2H_2_O, 0.02 g of H_3_BO_3_, 0.5 g of CaSO_4_·2H_2_O, 7 g of ZnCl_2_, 22 g of FeSO_4_·7H_2_O, 0.92 g of CoCl_2_·6H_2_O and 1 ml of concentrated H_2_SO_4_ (98%). All reagents were purchased from Soviquim (Santiago, Chile), unless otherwise stated.

The bioreactor operating conditions used were three temperatures (20, 25 and 30 °C), an airflow rate of 1.2 L min^−1^ (0.8 vvm), and a constant agitation speed of 700 rpm. The pH was kept at 5.0 by adding 15% vv^−1^ ammonium hydroxide (NH_4_OH) when required.

### Continuous cultivation

For chemostat operation, the bioreactor setup and medium were identical to those used in batch experiments. The feeding of fresh FM22 medium was initiated once the culture reached mid-exponential growth phase. The temperature was maintained constant at 20 °C, and the dilution rates (D) to be tested experimentally (0.025, 0.045 and 0.060 h^−1^) were determined based on the model simulations, ensuring that these values were at most 80% of the maximum specific cell growth rate determined in batch cultures at 20 °C. Periodic sampling was carried out after two complete volume changes, during residence times (τ) 3, 4 and 5 to confirm the stability of the steady state by evaluating cell biomass, carbon source concentration, and BGL1 enzymatic activity, where the standard deviation between measurements was below 5%. A DO control strategy was not required to be implemented (Fig. S2).

### Analytical methods

Biomass concentration was measured by optical density for flasks using a UV-Vis spectrophotometer (Jenway 6705, Vernon Hills, IL, USA) set at 600 nm, and by dry cell weight (DCW) for bioreactors. Glucose concentration was determined using the 3,5-dinitrosalicylic acid (DNS) method, whereas secreted protein quantification was performed using the Bradford method assay. The catalytic activity of β-glucosidase was quantified as described in Offei *et al*. (2022). All reagents were purchased from Sigma-Aldrich (St. Louis, MO, USA).

### Real-time quantitative PCR analyses

Two mL of culture broth (in duplicate) were collected in steady-state conditions and centrifuged at 7,500 rpm for 5 min (Hermle Z323K). The supernatant was discarded, and the cell pellet was resuspended in 500 µl of RNA later (Sigma-Aldrich, St. Louis, MO, USA) for storage at − 80 °C. Total RNA was extracted using the YeaStar RNA kit (Zymo Research, Irvine, CA, USA) according to the manufacturer’s instructions. Subsequently, reverse transcription was performed on the extracted RNA using the Affinity Script QPCR cDNA Synthesis kit (Agilent Technologies, Santa Clara, CA, USA) following the manufacturer’s guidelines. For gene expression analyses by RT-qPCR, 1 µl of cDNA was used in a 25 µl reaction volume with the Brilliant II SYBR Green qPCR Master Mix (Agilent Technologies, Santa Clara, CA, USA), following the manufacturer’s protocol. The relative expression level of genes *bgl1*,* GAPDH* (Velastegui et al. 2025), in addition to *HAC1*,* KAR2*,* PDI*, and *ERO1* (Huang et al. 2019) were estimated with the − 2∆∆Cq method (Pfaffl 2001) using the quantification cycle (Cq) values determined from genes *RSC-1* and *TAF10*, as reference genes (Besleaga et al. 2023). All primers were purchased from IDT (Coralville, IA, USA).

### Statistical analysis

Statistical analyses were performed using a one-way ANOVA test in GraphPad Prism v10. Differences were considered statistically significant at *p* < 0.05 (*), *p* < 0.005 (**), *p* < 0.0005 (***), and *p* < 0.0001 (****).

### Dynamic model

Preliminary analysis of the experimental data revealed a dynamic decoupling between the specific substrate uptake rate and the specific cell growth rate (Supplementary Figure S1), estimated by differentiating cubic smoothing splines fitted to substrate and biomass concentration data (Bayer et al. 2019). Based on this observation, a dynamic model was proposed to describe the evolution of biomass (* x*), protein (* P*), and substrate (* S*) concentration. Following the Droop quota framework (Droop 1968), we introduced an additional variable *(z)*, known as cell quota, to capture the experimentally observed lag between specific cell growth rate and specific substrate uptake. The model was formulated as a system of ordinary differential equations (ODEs) as follows:

1 $$\:\frac{dx}{dt}=\mu\:\left(z,T\right)x-m\left(T\right)x$$

2 $$\:\frac{dS}{dt}=\:-\:\rho\:(S,T)x$$

3 $$\:\frac{dz}{dt}=\:\rho\:(S,\:T)\:-\:\mu\:(z,\:T)z$$

4 $$\:\frac{dP}{dt}=\:q(z,T)x\:-\:{m}_{p}\left(T\right)P$$

where µ is the specific growth rate defined as:

5 $$\:\mu\:(z,T)={\mu}_{max}\left(T\right)\:\left(1-\frac{{z}_{min\:}}{z}\right)$$

with $\:{\mu}_{max}\left(T\right)$ the maximum specific growth rate and $\:{z}_{min}$ the minimum value allowed for * z* (known as subsistence cell quota). The term $\:m\left(T\right)$ corresponds to the specific decay rate of biomass. The term $\:\rho\:(S,T)$ is the substrate uptake rate defined as:

6 $$\:\rho\:(S,T)={\rho}_{max}\left(T\right)\:\frac{S}{S\:+\:{K}_{S}\:}$$

with $\:{\rho}_{max}\left(T\right)$ as the maximum uptake rate, and $\:{K}_{S}$ as the half-saturation constant. Finally, $\:q(z,T)\:$corresponds to the specific protein production rate, defined as:

7 $$\:q(z,T)\:=\:{q}_{max}\left(T\right)\:\left(1-\frac{{z}_{min\:}}{z}\right)$$

with $\:{q}_{max}\left(T\right)$ as the specific maximum protein production rate, and $\:{m}_{P}\left(T\right)$ as the protein degradation rate.

### Parameter fitting

Parameters $\:{\mu}_{max}$, * m*, $\:{m}_{P}$, $\:{\rho}_{max}$ and $\:{q}_{max}$, along with the initial conditions $\:{x}_{0}$, $\:{P}_{0}$, and $\:{S}_{0}$ were estimated independently for each temperature. Since * z* is not experimentally measurable, its scale and initial value are not well constrained by the available extracellular measurements. Our primary interest in this latent variable is to capture the delay between substrate uptake and growth rather than to determine its absolute value; we therefore adopted the following assumptions. The parameter $\:{z}_{min}$ as well as the initial condition $\:{z}_{0}$ were assumed to be equal to 1. This choice sets zero specific growth rate at inoculation (consistent with the lag phase observed experimentally) and corresponds to roughly a third of the substrate consumed per unit of biomass produced, estimated from the batch yield data, placing it within a physiologically reasonable range. Additionally, since the simultaneous estimation of $\:{\mu\:}_{max}$ and $\:{K}_{S}$ is known to be challenging due to strong parameter correlation, the value of $\:{K}_{S}$ is set to 0.5 g L^−1^. We denote the set of parameters to be estimated by $\:\theta\:=({\mu\:}_{max},\:m,\:{m}_{P},\:{\rho}_{max},{q}_{max}\:{x}_{0},\:{P}_{0},\:{S}_{0})$.

The parameters $\:\theta\:$ were estimated by minimizing the normalized sum of squared residuals (NSSR) between the experimental measurements and model predictions. The NSSR is defined as:

8 $$\:J\left(\theta\:\right)=\sum\limits_{y\varepsilon \{x,S,P\}}^{}\sum\limits_{i=1}^{n}{\left(\frac{{y}_{i,\:obs}-{y}_{i}\left(\theta\:\right)}{\underline{y}_{obs} }\right)}^{2}$$

where $\:{y}_{i,\:obs}$ and $\:{y}_{i}\left(\theta\:\right)$ are the observed value and predicted values of the state variable $\:y\:$ at time *t*_*i*_, respectively, ${\underline{y}_{obs}}$ denotes the mean of the observations for the state variable * y*, and * n* is the total number of sampling times.

All simulations and parameter estimations were performed in Python. Model predictions were obtained by numerically solving the system of ordinary differential equations (Eqs. 1 to 4) using an explicit Runge-Kutta method of order 5(4) (RK45), as implemented in *solve_ivp* from the *SciPy* library. Parameter estimation was performed by solving a nonlinear least squares problem using the least_squares routine implemented in the lmfit library (Newville et al. 2014) which employs a trust-region reflective algorithm and allows the enforcement of bound constraints on the parameters. Non-negativity constraints were imposed on all fitted parameters to ensure physical plausibility. To quantify the uncertainty associated with the fitted parameters, the asymptotic errors were computed from the estimated covariance matrix returned by the nonlinear least-squares procedure. Each parameter is reported as estimate ± asymptotic error, together with the corresponding relative uncertainty (100×SE/|estimate|).

## Results and discussion

### Cell growth and protein expression in batch cultures

**Fig. 1:**
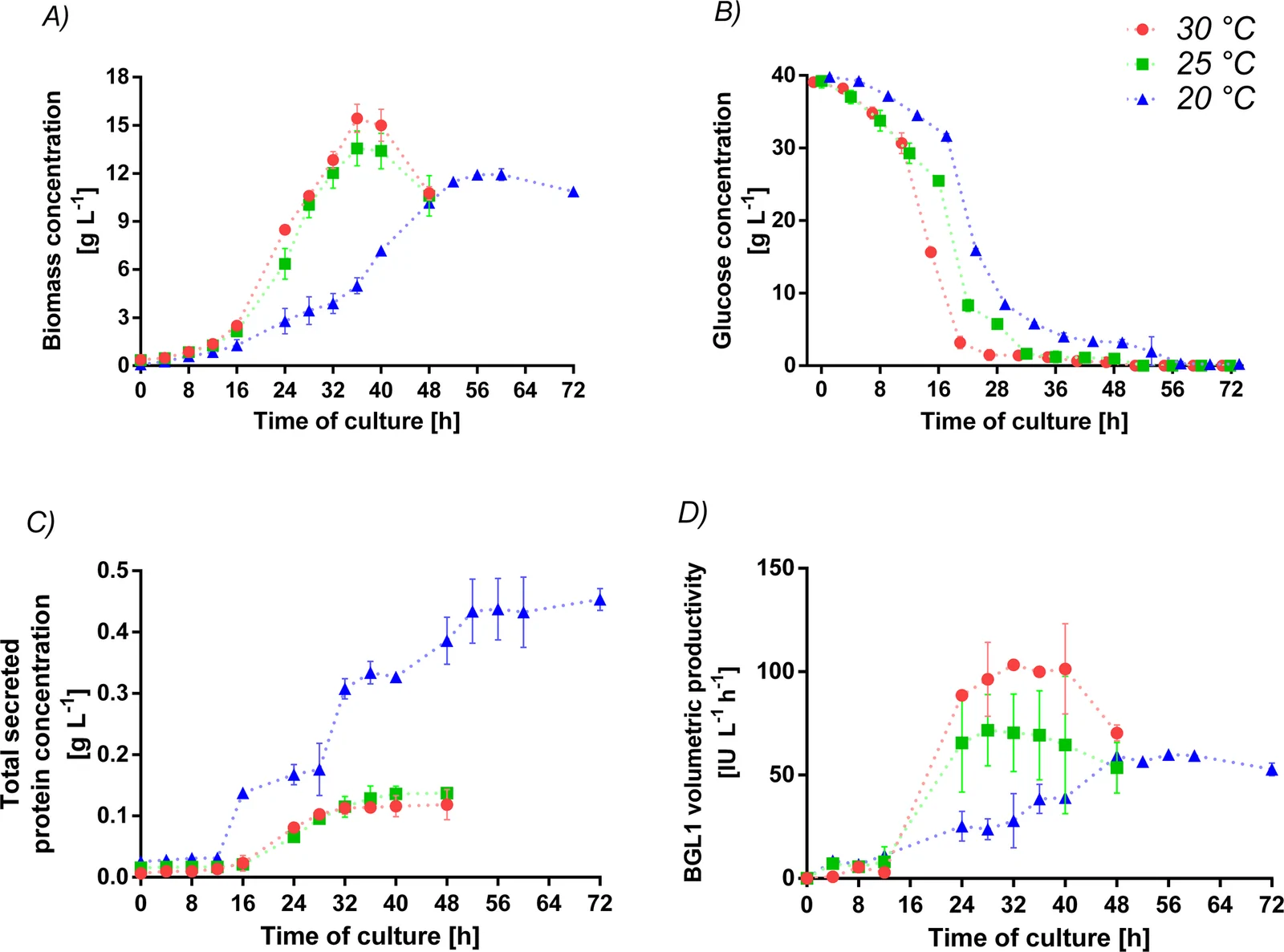
Biomass generation (**A**), glucose consumption (**B**), total secreted protein (**C**) and BGL1 volumetric productivity (**D**) of *K. phaffii* S467 grown in bioreactors at 20, 25 and 30 °C. All data points represent the average of 2 biological replicates and 2 technical replicates for each, with standard deviation bars indicated

Biomass, total secreted protein and BGL1 enzymatic activity were evaluated at 20, 25 and 30 °C in batch cultures of *K. phaffii* S467 (Fig. 1). The biomass concentration reached was markedly impacted by the temperature change, with a 50% decrease in maximum specific growth rate (µ_max_) (Table 1) when the cultivation temperature was reduced to 20 °C, whereas at 25 °C the cultures exhibited a similar cell growth profile than what was observed at 30 °C.

Expectedly, the glucose showed a slower decrease over time at 20 °C (Fig. 1B), whereas the specific consumption rate at 25 °C was comparable to the rate observed at 30 °C. The total secreted protein concentration was measured as an indicator of recombinant protein expression, since it has been consistently reported that *K. phaffii* secretes a low number of native proteins (Zhu et al. 2019; Burgard et al. 2020), which results in the secreted fraction to be composed mostly by the recombinant protein of interest. Figure 1C shows a 4-fold increase in secreted protein at 20 °C, which is in agreement with previous studies reporting enhanced recombinant protein expression at lower temperature at the expense of specific growth rate (Li et al. 2001; Cos et al. 2006; Dragosits et al. 2009). However, despite this increment, the highest BGL1 enzymatic activity in the supernatant, hereafter referred to as BGL1 volumetric productivity (Fig. 1D), was achieved at 30 °C, largely due to the noticeable impact of lower temperature on biomass production.

### Parameter fitting and simulations

The use of a Droop-based mathematical model was motivated by the dynamic decoupling between specific substrate uptake rate and specific growth rate observed in the preliminary analysis of the experimental data (Fig. S1). Classical unstructured models, which typically combine Monod-type kinetics with the assumption of a constant biomass yield, impose a proportional relationship between the specific growth rate and the specific substrate consumption rate. However, the estimated specific rates indicate that this proportionality does not hold throughout the culture, suggesting that classical growth models are not the best fit to describe the observed dynamics.

**Fig. 2:**
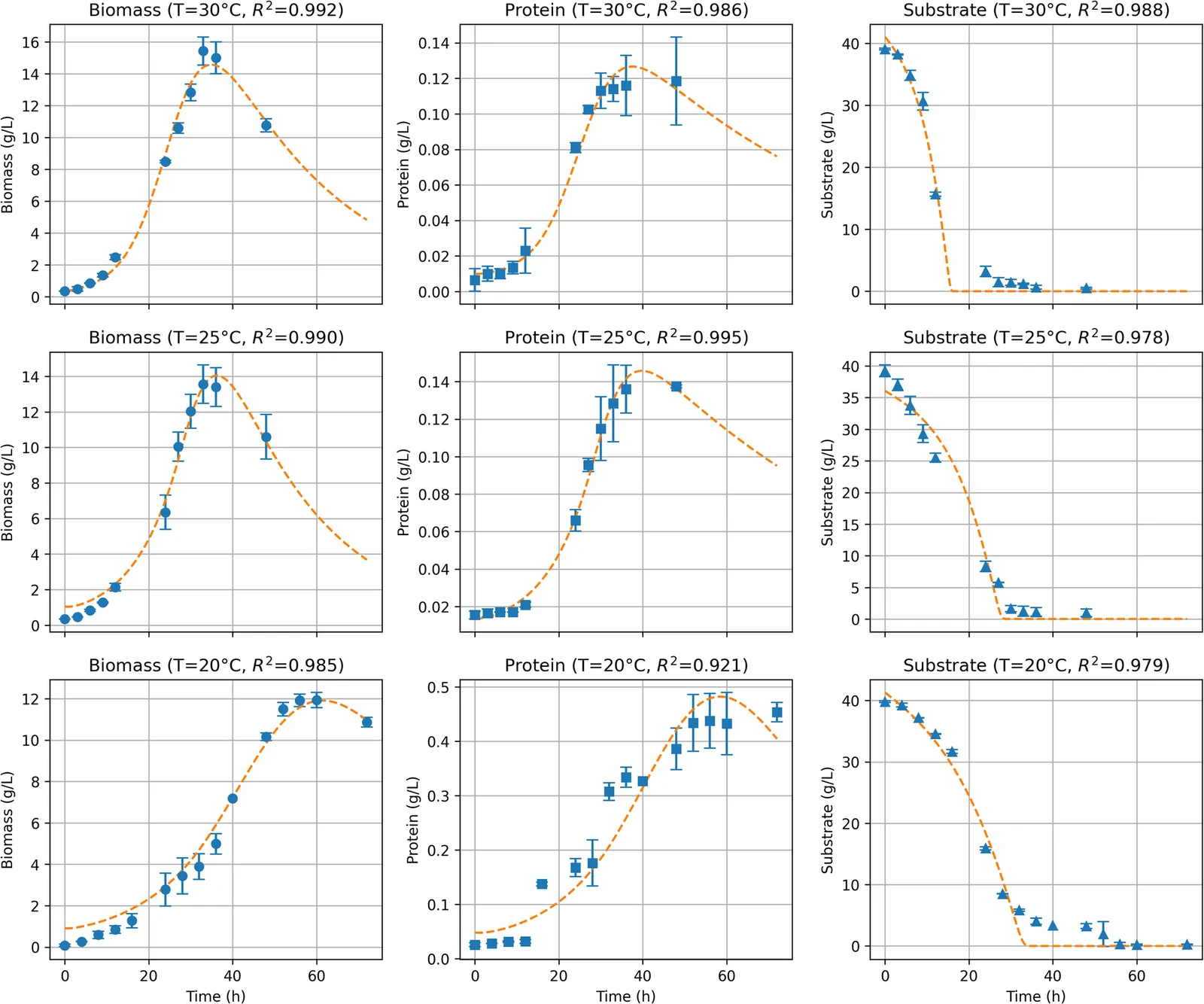
Experimental data (dots) and model predictions (dashed lines) for biomass, total secreted protein, and substrate concentrations as a function of time at three cultivation temperatures (20, 25, and 30 °C). Each row corresponds to a different temperature, while columns show biomass concentration, total secreted protein concentration, and glucose concentration, respectively. Error bars represent experimental uncertainty, and the coefficient of determination R^2^ reported in each panel quantifies the fit between model predictions and experimental measurements

The experimental data fitting to a Droop-based model (Fig. 2) shows good agreement between the data from the batch cultures and the model predictions, with coefficients of determination (R²) ranging from 0.921 to 0.995 across all variables and temperatures (Supplementary Table S5). The lowest value corresponds to total secreted protein at 20 °C, consistent with the lower protein titres, and correspondingly lower signal-to-noise ratio, obtained at this temperature. Residual and observed-versus-predicted diagnostics (Supplementary Figure S6) show no systematic trend with time for any variable, and relative bias remains below 7% of the mean observed value in all cases, indicating that the model captures the batch dynamics without a consistent tendency to over- or under-predict any of the three state variables. Moreover, the parameter estimates reported in Table 1 support a satisfactory practical identifiability of the model (Wieland et al. 2021), since most parameters exhibit low to moderate relative uncertainty across temperatures (typically < 20% for *µ*_*max*_, *m*, and *q*_*max*_), a pattern confirmed by profile likelihood analysis (Wieland et al. 2021) showing well-defined, bounded profiles for these parameters (Supplementary Figure S3). Larger uncertainty is observed for m_P_, which is expected because only a limited number of measurements are directly informative of product degradation (protein decay), making this parameter less identifiable from the available batch time-series data. Multi-start optimization confirmed that the reported estimates correspond to a unique, well-defined global optimum rather than a local minimum dependent on the initial guess (Supplementary Figure S4). The Droop-based model also outperformed a conventional Monod-based model fitted to the same data (Supplementary Figure S5), consistent with the dynamic decoupling between substrate uptake and growth described above. The parameter estimates reported in Table 1 were robust to the limited number of biological replicates: fitting the model to the pooled individual measurements, or to each replicate independently, reproduced the reported values within 2.5% in the worst case (Supplementary Table S2), confirming that averaging the replicates prior to fitting did not bias the results. Finally, a sensitivity analysis confirmed that the fitted kinetic parameters and the model-based conclusions were robust to the fixed values of $\:{K}_{S}$ and $\:{z}_{0}$, changing by less than 3% when these constants were varied over a wide range; releasing $\:{z}_{min}$ or $\:{z}_{0}$ as free parameters did not improve the fit meaningfully and produced physiologically inconsistent behaviour, supporting the decision to fix them (Supplementary Tables S3 and S4).

**Table 1:** Estimated parameter values obtained from the Droop-based dynamic model for strain *S467* cultivated in batch mode at different temperatures. For each parameter, the table reports the fitted estimate as mean ± asymptotic error

Temperature	µ_max_ [h^−1^]	*m* [×10^−2^ h^−1^]	*q*_max_ [×10^−3^ h^−1^]	*m*_*P*_ [×10^−2^ h^−1^]	ρ_max_ [h^−1^]
30 °C	0.201 ± 0.016	3.49 ± 0.17	1.37 ± 0.14	1.72 ± 0.60	2.30 ± 0.66
25 °C	0.217 ± 0.015	4.41 ± 0.28	1.57 ± 0.17	1.54 ± 0.68	0.381 ± 0.040
20 °C	0.0978 ± 0.0078	2.44 ± 0.20	4.95 ± 0.96	3.7 ± 1.3	0.587 ± 0.082

µ_max_: maximum specific growth rate; *m*: specific decay rate of biomass; *q*_max_: specific maximum protein production rate; *m*_P_: protein degradation rate; ρmax: maximum substrate uptake rate

**Fig. 3:**
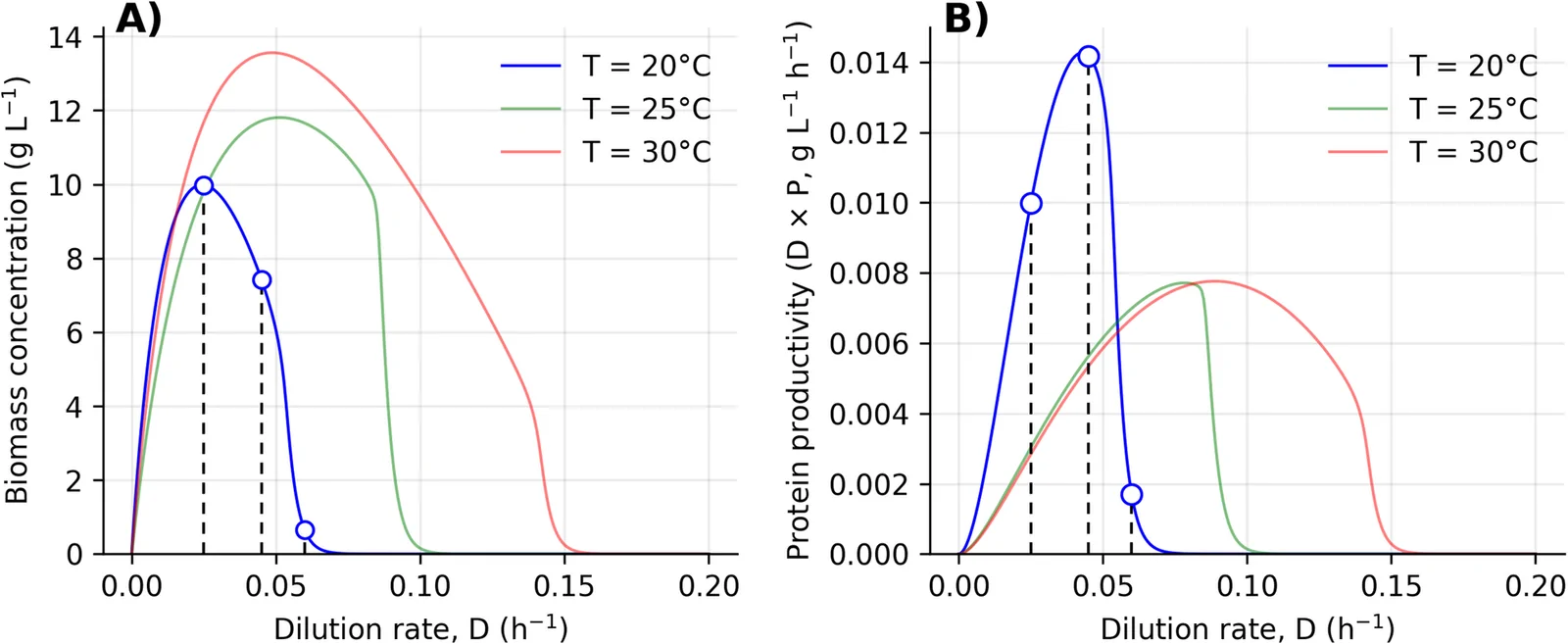
Simulated steady-state biomass concentration (**A**) and protein productivity Q_P_ (**B**) as a function of the dilution rate for continuous cultures at 20, 25, and 30 °C. Solid lines correspond to model predictions at each temperature. Vertical dashed lines indicate selected dilution rates for experimental evaluation (D = 0.025, 0.045, and 0.06 h^−1^); round markers denote the corresponding values on the 20 °C curve

Using the Droop-based model fitted to experimental data at 20, 25, and 30 °C (Table 1), we then extended the analysis to continuous cultivation by simulating a chemostat across the full range of admissible dilution rates. For each temperature and dilution rate (D*)*, the system was integrated until reaching a steady-state regime, and the resulting steady-state biomass (x) and total secreted protein concentration (P*) were recorded. The steady-state volumetric productivity of total secreted protein was then computed as Q_P_= D*P and used to compare operating conditions across temperatures (Fig. 3).

The simulations indicated that the maximum volumetric productivity of total secreted protein was achieved at 20 °C with a *D* = 0.045 h^−1^ (Fig. 3B). Although this condition yields the lowest steady-state biomass among the temperature range evaluated (Fig. 3A), it predicts the highest protein productivity, consistent with the fitted kinetics showing a comparatively higher production capacity at low temperature (notably a larger *q*_*max*_ at 20 °C, as shown in Table 1). This predicted optimum was found to be robust to the value assumed for the half-saturation constant K_S_: the predicted optimal dilution rate at 20 °C changed by less than 2% when K_S_ was varied over two orders of magnitude (Supplementary Table S3).

Based on these considerations, three dilution rates were selected for experimental evaluation in continuous cultures at 20 °C. As illustrated in Fig. 3, the rates predicted to yield the maximum biomass concentration and the highest secreted protein productivity, 0.025 h^−1^ and 0.045 h^−1^, respectively, were chosen. Additionally, a third dilution rate (0.06 h^−1^) was evaluated, representing the highest value feasible according to the simulated critical dilution rate.

### Experimental evaluation in chemostat cultivation

**Fig. 4:**
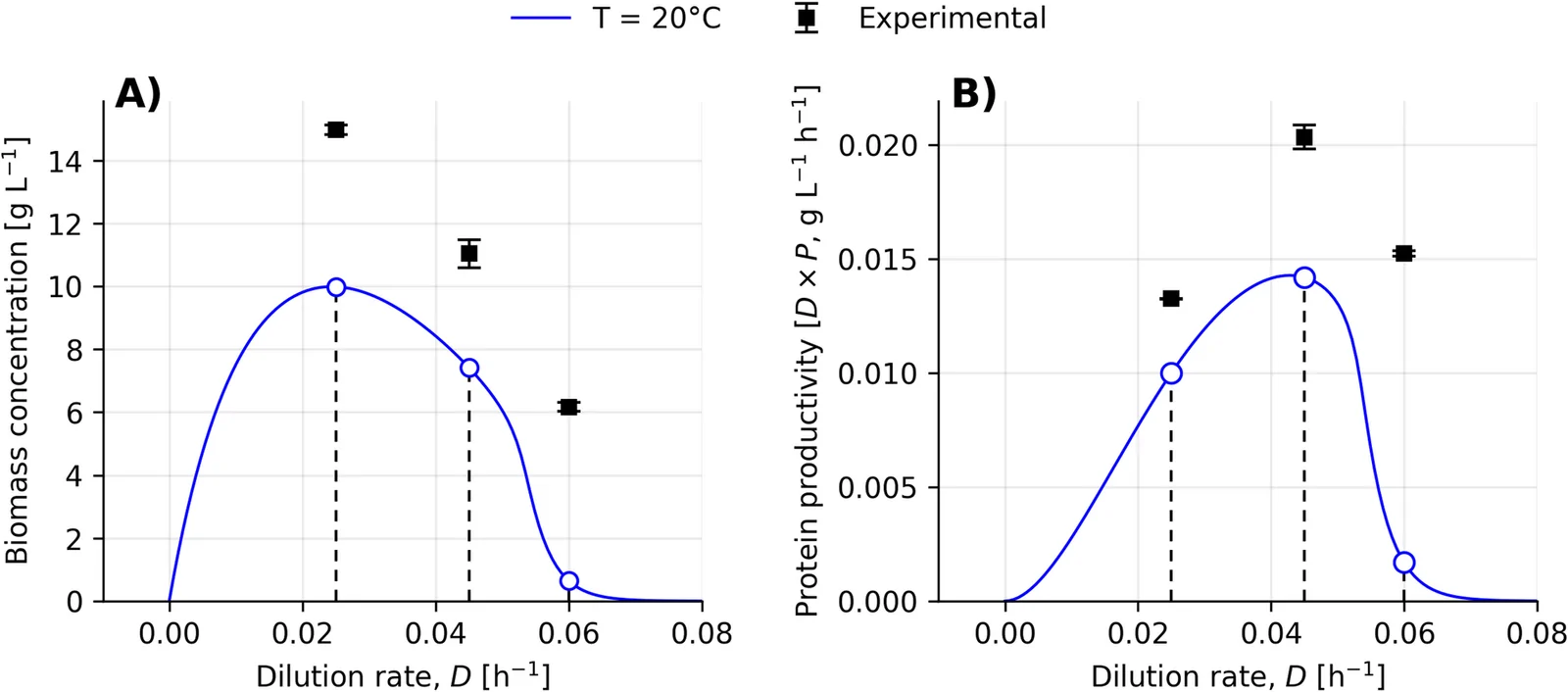
Experimental steady-state biomass concentration (**A**) and total secreted protein productivity (**B**). Black squares correspond to experimental results from three different residence times, and the average value is displayed with its corresponding standard deviation. Solid blue lines correspond to the model predictions at each dilution rate, with white circles highlighting the dilution rates evaluated

The operational conditions suggested by the simulation were tested in chemostat cultures. The biomass concentration decreased with increasing D, reaching a concentration of 11 g L^−1^ at D = 0.045 h^−1^, which is 23% higher than the estimated value predicted by the model (Fig. 4A).

Q_P_ effectively reached a maximum value at the dilution rate of 0.045 h^−1^ (Fig. 4B) among the conditions tested, consistent with the trend predicted by the simulations. However, the model underestimated the productivity achieved, as the experimental Q_P_ was approximately 36% higher than the predicted value for the three dilution rates evaluated (Table S1). While an increase in specific growth rate might imply higher productivity, the maximum secreted protein productivity was achieved at D = 0.045 h^−1^ among the experimentally evaluated dilution rates, consistent with the model-predicted productivity maximum.

The model’s underestimation of biomass and total secreted protein concentration compared to the experimental chemostat results (averaging 36.5% and 33.7%, respectively) suggests that the parameters derived from batch cultures are limited from fully capturing the cellular physiology under sustained glucose limitation and pseudo-steady-state conditions. This discrepancy contrasts with studies where the validation occurred within operational frameworks closer to the model calibration. For example, Dahiya *et al*. employed a kinetic model that reproduced biomass with high fidelity and protein accumulation with a close approximation within a single high-cell-density process (Dahiya et al. 2026). Similarly, Luna-Flores *et al*. observed that parameters from batch and continuous cultures accurately predicted fed-batch performance (Luna-Flores et al. 2023). Nevertheless, the literature highlights that transitioning between cultivation regimes often affects process variables disproportionately, with protein production exhibiting greater sensitivity than biomass growth. D’anjou and Daugulis, with continuous stirred-tank reactor data, effectively predicted fed-batch biomass yet underestimated recombinant protein concentration (D’anjou and Daugulis 2001). Likewise, Bolmanis *et al*. achieved a good overall fit for biomass and product, even though underestimating late-stage biomass and residual methanol concentration, along with certain productivity peaks (Bolmanis et al. 2022). Alternatively, Boojari *et al*. emphasized that modeling is a useful tool for defining operational strategies rather than precise final value predictions (Boojari et al. 2023). Collectively, these findings suggest that the observed discrepancy is not merely a model limitation, but rather further evidence that shifting from a transient batch to a continuous chemostat regime is not a linear process.

Despite the underestimation observed in the model, the experimental trend for biomass and total secreted protein production remained consistent across the evaluated dilution rates. To quantify this relationship, a correlation coefficient was calculated for both datasets, yielding values of 0.97 for biomass and 0.77 for total secreted protein concentrations. While these results indicate a higher fit for the biomass concentration data, the strong positive correlation (r^2^ > 0.7) maintained for both parameters supports the model as a useful tool for delineating the experimental conditions that can be explored to maximize the bioprocess performance for strain S467.

**Fig. 5:**
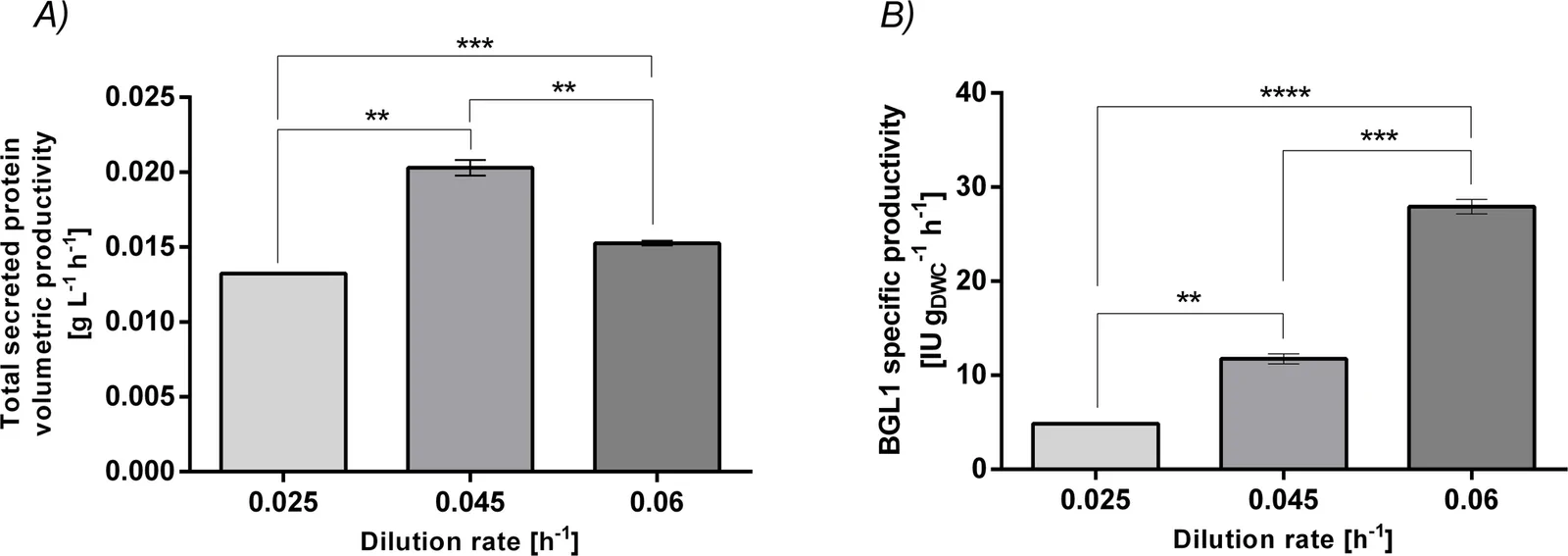
Volumetric productivity of total secreted protein (**A**) and specific activity of β-glucosidase (**B**) under steady-state conditions at different dilution rates. Sampling was carried out at three different residence times, and the average value is displayed with its corresponding standard deviation. Differences were considered statistically significant at *p* < 0.005 (**), *p* < 0.0005 (***), and *p* < 0.0001 (****)

When analyzing more specifically the recombinant protein expression as a function of enzymatic activity, BGL1 specific productivity (q_BGL1_) showed an incremental trend as dilution rates increased, with the highest specific enzymatic activity detected at the highest dilution rate (Fig. 5). On the contrary, we observed that the intermediate dilution rate tested improved the total secreted protein volumetric productivity, possibly due to a balanced trade-off between biomass concentration and specific secretion rate. This interplay has been previously reported for *K. phaffii* (Torres et al. 2019) where the authors used a predictive genome-scale metabolic model based on glucose-limited chemostat data, finding that optimal growth and protein production lie in opposite operational regions, highlighting the natural trade-off from these metabolic tasks. The improved specific enzymatic activity measured at higher D could be attributed to faster-growing cells secreting BGL1 with higher enzymatic activity, likely due to improved folding, reduced proteolysis, and/or more efficient processing through the secretory pathway. A recent study using a methanol-free bi-directional promoter system in continuous cultivation monitored biomass-specific enzymatic activity at different dilution rates and observed that the recombinant enzyme’s performance was strongly dilution-rate dependent. This work reported lower secretion/activity at low D and investigated higher D values to improve enzyme production, which further illustrates that enzymatic activity and dilution rate can be coupled in a non-trivial manner (Besleaga et al. 2025).

At higher D the residual carbon source often increases, and cells can experience less severe nutrient limitation. Consequently, folding and post-translational protein processing could possibly occur in more optimal conditions than under stronger nutrient limitation (lower D), where cellular maintenance requirements consume a larger fraction of resources. The culture at D = 0.06 h^−1^ exhibited increased residual glucose concentration and glucose uptake rate (Table S1), which would be in agreement with the latter. In *K. phaffii*, growth rate has been previously shown to strongly influence cellular physiology, stress responses, protein synthesis, and secretion pathways (Canales et al. 2015). Transcriptomic analyses reported by Rebnegger *et al*. (Rebnegger et al. 2014) demonstrated that increasing specific growth rate alters the expression of genes involved in protein synthesis, secretion, and stress responses, implying that the biological activity, and not only the concentration, of recombinant proteins could change with growth rate. We hypothesize that a higher dilution rate could also result in faster washout of extracellular proteases relative to their production rate, decreasing the exposure time of secreted BGL1 to degrading conditions, which could generate higher activity per biomass. Proteolytic degradation by endogenous proteases is a recognized limitation for recombinant protein production in *K. phaffii*, and recent evidence has identified specific endogenous proteases capable of directly affecting recombinant protein stability and final product titers (Feng et al. 2026).

At intermediate D, a higher secretion flux might be plausible, which could compromise the fraction of active vs. inactive enzyme forms, providing overall reduced catalytic activity. In addition, the glycosylation pattern could also vary, contributing to varying enzymatic activity for different D. Earlier reports of expression of BGL1 in *K. phaffii* have shown that, indeed, BGL1 is glycosylated; however, the glycosylation profile was not described (Hong et al. 2007).

**Fig. 6:**
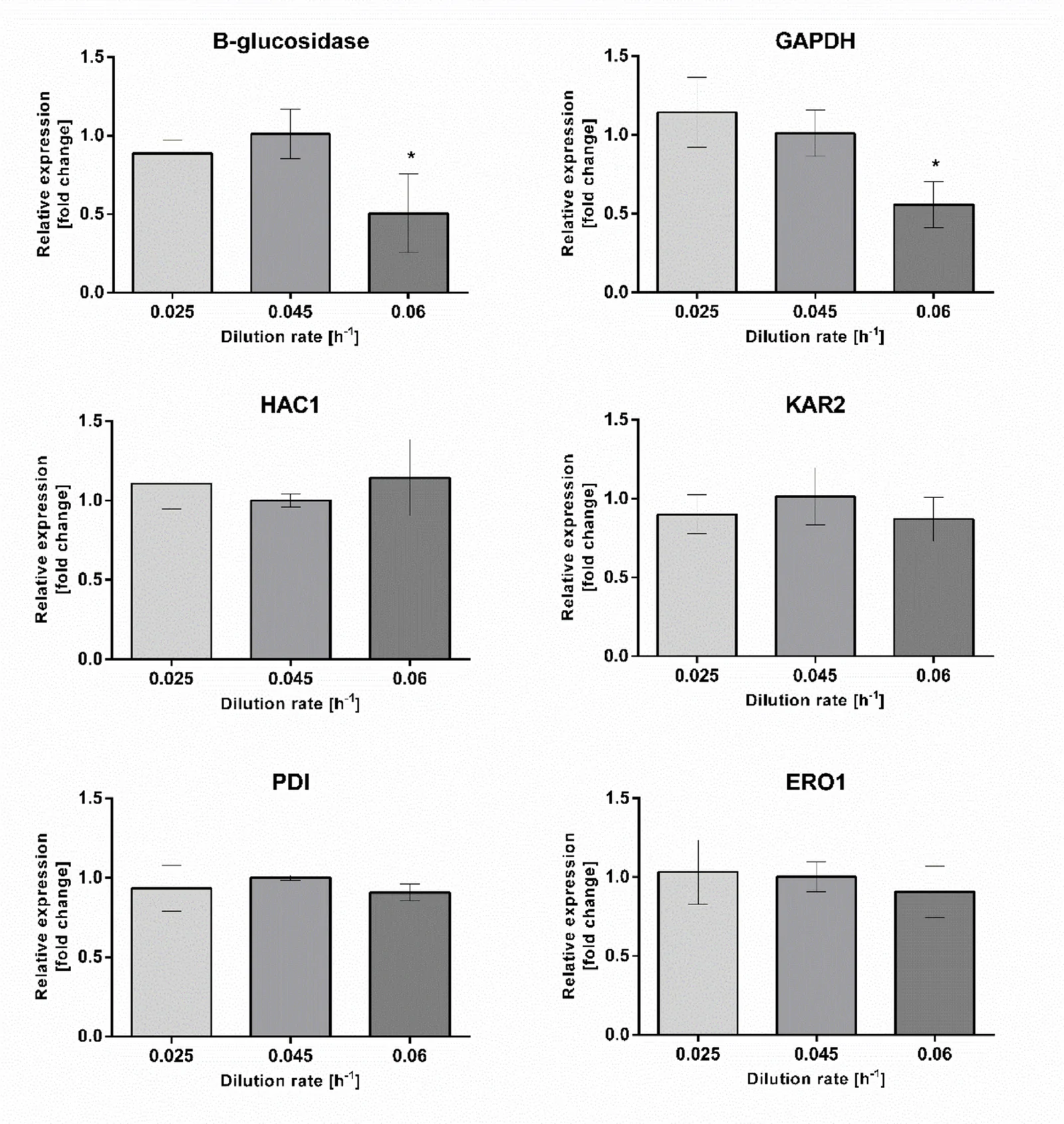
Transcription levels of the gene *bgl1* encoding β-glucosidase under control of the *pGAP* promoter, the native gene *GAPDH* under control of the *pGAP* promoter, and genes associated with ER stress (*HAC1*,* KAR2*,* PDI* and *ERO1*)

Lastly, ER stress is sensed via the transcriptional activator *HAC1*, which activates the expression of genes such as *KAR2*, encoding molecular chaperones that assist in protein folding; *PDI*, encoding a protein disulfide isomerase, which rearranges disulfide bonds in misfolded proteins; and *ERO1*, encoding an oxidase that reoxidizes the protein disulfide isomerase, enabling its reuse (Vanz et al. 2014; Huang et al. 2019). The gene expression level of these markers, alongside the relative expression of *bgl1* was evaluated (Fig. 6) showing no significant transcriptional changes under the evaluated steady-state conditions.

In addition, the relative expression of *bgl1* decreased slightly at higher D, which could suggest that under conditions of higher specific growth rate enhanced efficiency in post-translational mechanisms or in the secretory pathway could occur, promoting the accumulation of a larger fraction of active enzyme, despite a lower mRNA abundance.

## Conclusion

In this study, we combined experimental characterization with dynamic mathematical modeling to investigate the effect of cultivation temperature on cell growth and recombinant β-glucosidase production in *K. phaffii*. Lowering the cultivation temperature to 20 °C increased recombinant protein secretion at the expense of biomass formation, revealing a clear trade-off between cell growth and protein productivity. To quantitatively describe these dynamics, we developed a model that takes into account the delayed coupling between substrate uptake and biomass formation, which captured the main experimental trends and provided a reliable framework for exploring in more detail the effect of cell growth rate in continuous cultivation.

Model-based simulations identified chemostat conditions of 20 °C and a dilution rate of 0.045 h^−1^ as the optimal operating condition to maximize volumetric total secreted protein productivity. These conditions were subsequently evaluated experimentally, providing a close fit to the simulation; however, additional dilution rates in the vicinity of the optimum simulated value would be required to experimentally determine the optimum dilution rate with greater precision. Moreover, the highest BGL1 enzymatic activity was observed at the highest dilution rate, despite lower *bgl1* transcript abundance, which suggests that specific growth rate could influence other recombinant protein traits through post-transcriptional or post-translational mechanisms rather than transcriptional regulation alone. These findings highlight that optimizing recombinant protein production requires consideration of both the secretion yield and the generation of biologically active proteins.

Beyond identifying improved operating conditions, this work demonstrates the value of integrating experimental characterization with dynamic modeling to guide rational bioprocess optimization. While hypothermia has been previously reported as a useful strategy to improve recombinant protein production, quantitative frameworks capable of predicting the combined effects of temperature and growth rate on process performance remain limited. Furthermore, predictive approaches can reduce reliance on empirical optimization while offering insights into the interplay between cultivation conditions, recombinant protein secretion, and inherent protein traits. These findings provide a framework that may be evaluated for other recombinant proteins, strain backgrounds and cultivation conditions with the ultimate goal of supporting the development of more efficient and robust bioprocesses.

## Supplementary Information

Below is the link to the electronic supplementary material.

Supplementary Material 1

## Data Availability

The datasets supporting the conclusions of this article are available in the GitHub repository https://github.com/carlosmvond/Rocco_et_al_2026. The codes in Python used for model implementation, parameter estimation, and simulation are provided in the same repository under the MIT License.

## Abbreviations

BGL1: β-Glucosidase
D: Dilution rate (h^−1^)
DCW: Dry cell weight
ER: Endoplasmic reticulum
*GAPDH*: Glyceraldehyde 3-phosphate dehydrogenase gene
IU: International activity units (mM min^−1^)
K_S_: Half-saturation constant
*m*: Specific decay rate of biomass (h ^−1^)
*m*_P_: Protein degradation rate (h^−1^)
OD_600_: Optical density at 600 nanometers
*P*: Product concentration (g L^−1^)
*q*_max_: Maximum protein production rate (g g^−1^ h^−1^)
Q_P_: Protein volumetric productivity (g L^−1^ h^−1^)
q_P_: Protein specific productivity (g g^−1^ h^−1^)
*Ρ*: Substrate uptake rate (h^−1^)
ρ_max_: Maximum uptake rate (g substrate g^-1^ biomass h^-1^)
*S*: Substrate concentration (g L^−1^)
τ: Hydraulic residence time (h)
µ: Specific growth rate (h^−1^)
µ_max_: Maximum specific growth rate (h^−1^)
*x*: Biomass concentration (g L^−1^)
*z*: ell quota (g substrate g^−1^ biomass)
Y_x/s_: Biomass yield on substrate (g biomass produced g^−1^ substrate consumed)
