# Supplementary material for: Integrating dynamic modeling and continuous cultivation to enhance recombinant β-glucosidase production in Komagataella phaffii
Source: Bioresour Bioprocess. 2026 Oct 15;13(1):151. doi: 10.1186/s40643-026-01147-9 (PMC13672270; doi:10.1186/s40643-026-01147-9)
Supplement: Supplementary file 1 [file 40643_2026_1147_MOESM1_ESM.docx]

**Supplementary information**

**Integrating dynamic modeling and continuous cultivation to enhance recombinant β-glucosidase production in *Komagataella phaffii***

Eduardo Rocco-Martínez^1†^, Johan Quezada^1†^, Julio Berrios^1,3^, Kenneth H. Wolfe^2^, Alvaro Díaz-Barrera^1,3^, Carlos Martínez^1^, Stephanie Braun-Galleani^1,3^*

**Table S1.** Summary of kinetics and productivity parameters obtained from chemostat cultures at 20°C at different dilution rates evaluated in steady state. Values are an average from 3 samples collected during the steady state spanning 2-3 residence times.

| **Dilution rate** | **Biomass concentration** | **Total protein productivity** | **BGL1 specific productivity** | **Glucose concentration** | **Glucose uptake rate** |
| --- | --- | --- | --- | --- | --- |
| **(h^-1^)** | **(g L^-1^)** | **(g_prot_ L^-1^ h^-1^)** | **(IU g_DCW_^-1^ h^-1^)** | **(g L^-1^)** | **(g_glu_ g_DWC_^-1^ h^-1^)** |
| 0.025 | 15.0±0.12 | 0.0132±2E-05 | 4.50 ± 0.05 | 0.166±5E-05 | 0.066±0.0008 |
| 0.045 | 11.0±0.18 | 0.0203±1E-04 | 11.15 ± 0.05 | 0.281±3E-04 | 0.161±0.0069 |
| 0.06 | 6.17±0.14 | 0.0152±1E-05 | 25.96 ± 0.06 | 1.028±7E-04 | 0.376±0.0085 |

**Table S2.** Replicate-level fitting of the kinetic model.

With n = 2 bioreactor cultures per temperature, fitting the model only to the replicate-averaged curve could mask replicate-to-replicate variability or bias the parameter estimates. To address this, the model was additionally fitted (i) to all individual measurements of both replicates pooled together, without averaging, and (ii)–(iii) to each biological replicate independently. The four resulting sets of estimates are compared below.

***Mean-curve fit:*** the approach used in Table 1 of the manuscript: the two replicates are averaged at each sampling time first, and the model is fitted once to that averaged curve.

***Pooled replicates:*** the model is fitted once to all individual data points of both replicates together, with no averaging beforehand.

***Replicate 1 / Replicate 2:*** the model is fitted separately to each bioreactor run, using only the data from that single culture.

***Half-range (n = 2):*** |replicate 1 − replicate 2| / 2. With only two biological replicates, a conventional standard deviation (n − 1 = 1) is not informative; the half-range is the direct dispersion measure between the two runs, equal to the distance of either replicate from their mean.

***Dev. mean vs pooled:*** the relative difference between the mean-curve fit and the pooled-replicate fit, 100 × |pooled − mean| / |mean|, i.e. whether averaging replicates before fitting biases the estimate.

| **T (°C)** | **Param.** | **Units** | **Mean-curve**  **fit** | **Pooled**  **replicates** | **Rep. 1** | **Rep. 2** | **Half-range**  **(n = 2)** | **Dev. mean**  **vs pooled** |
| --- | --- | --- | --- | --- | --- | --- | --- | --- |
| **30** | μmax | h⁻¹ | 0.201 | 0.201 | 0.198 | 0.202 | 0.00202 | 4.3×10⁻⁴% |
|  | m | h⁻¹ | 0.0349 | 0.0349 | 0.0358 | 0.0341 | 8.60×10⁻⁴ | 7.8×10⁻⁵% |
|  | qmax | h⁻¹ | 0.00137 | 0.00137 | 0.00136 | 0.00142 | 3.16×10⁻⁵ | 8.0×10⁻⁵% |
|  | mP | h⁻¹ | 0.0172 | 0.0172 | 0.00894 | 0.0277 | 0.00938 | 0.002% |
|  | ρmax | h⁻¹ | 2.30 | 2.30 | 2.24 | 2.30 | 0.0327 | 0.002% |
|  | x0 | g L⁻¹ | 0.369 | 0.369 | 0.397 | 0.355 | 0.0211 | 0.002% |
|  | P0 | g L⁻¹ | 0.00990 | 0.00990 | 0.00466 | 0.0153 | 0.00531 | 0.001% |
|  | S0 | g L⁻¹ | 41.0 | 41.0 | 40.9 | 41.1 | 0.0824 | 1.5×10⁻⁵% |
| **25** | μmax | h⁻¹ | 0.217 | 0.217 | 0.211 | 0.225 | 0.00674 | 1.1×10⁻⁴% |
|  | m | h⁻¹ | 0.0441 | 0.0441 | 0.0476 | 0.0409 | 0.00333 | 2.5×10⁻⁵% |
|  | qmax | h⁻¹ | 0.00157 | 0.00157 | 0.00134 | 0.00180 | 2.29×10⁻⁴ | 1.3×10⁻⁴% |
|  | mP | h⁻¹ | 0.0154 | 0.0154 | 0.00916 | 0.0204 | 0.00563 | 5.2×10⁻⁵% |
|  | ρmax | h⁻¹ | 0.381 | 0.381 | 0.419 | 0.351 | 0.0340 | 7.7×10⁻⁴% |
|  | x0 | g L⁻¹ | 1.08 | 1.08 | 1.05 | 1.08 | 0.0153 | 0.001% |
|  | P0 | g L⁻¹ | 0.0137 | 0.0137 | 0.0143 | 0.0132 | 5.83×10⁻⁴ | 2.5×10⁻⁴% |
|  | S0 | g L⁻¹ | 36.0 | 36.0 | 36.9 | 35.0 | 0.943 | 5.5×10⁻⁶% |
| **20** | μmax | h⁻¹ | 0.0978 | 0.0981 | 0.104 | 0.0959 | 0.00388 | 0.290% |
|  | m | h⁻¹ | 0.0244 | 0.0244 | 0.0254 | 0.0238 | 8.06×10⁻⁴ | 0.145% |
|  | qmax | h⁻¹ | 0.00495 | 0.00487 | 0.00385 | 0.00522 | 6.83×10⁻⁴ | 1.44% |
|  | mP | h⁻¹ | 0.0374 | 0.0365 | 0.0244 | 0.0387 | 0.00714 | 2.49% |
|  | ρmax | h⁻¹ | 0.587 | 0.590 | 0.655 | 0.557 | 0.0490 | 0.453% |
|  | x0 | g L⁻¹ | 0.927 | 0.919 | 0.754 | 1.01 | 0.129 | 0.827% |
|  | P0 | g L⁻¹ | 0.0481 | 0.0488 | 0.0668 | 0.0394 | 0.0137 | 1.52% |
|  | S0 | g L⁻¹ | 41.2 | 41.2 | 40.8 | 41.4 | 0.274 | 0.036% |

**Table S3.** Sensitivity of the fitted kinetic parameters and the predicted optimal chemostat dilution rate (D_opt) to the fixed values of the half-saturation constant (K_S) and the initial cell quota (z0, with zmin = 1 g g⁻¹ held fixed). For each temperature, the model was refitted with K_S or z0 held at each listed value while all other parameters were estimated. J is the value of the normalised objective function at the optimum (Eq. 8); m, qmax and mP are reported ×10⁻², ×10⁻³ and ×10⁻² h⁻¹, respectively. Across the entire range tested, the kinetic parameters of primary biological interest (μmax, m, qmax, mP) change by less than 3% at every temperature. D_opt is essentially insensitive to K_S at 30 and 20 °C (change ≤ 2%) but shows moderate sensitivity at 25 °C (change ≈ 15% across the tested range); it is insensitive to z0 at all three temperatures (change ≤ 2%).

| **T**  **(°C)** | **Fixed**  **constant** | **Value** | **J** | **μmax**  **[h⁻¹]** | **m**  **[×10⁻² h⁻¹]** | **qmax**  **[×10⁻³ h⁻¹]** | **mP**  **[×10⁻² h⁻¹]** | **ρmax**  **[h⁻¹]** | **D_opt**  **[h⁻¹]** |
| --- | --- | --- | --- | --- | --- | --- | --- | --- | --- |
| **30 °C** | *K_S* | 0.05 | 0.2953 | 0.2007 | 3.49 | 1.372 | 1.72 | 2.261 | 0.0891 |
|  |  | 0.10 | 0.2954 | 0.2007 | 3.49 | 1.372 | 1.72 | 2.265 | 0.0891 |
|  |  | 0.25 | 0.2959 | 0.2007 | 3.49 | 1.372 | 1.72 | 2.277 | 0.0890 |
|  |  | 0.50 | 0.2965 | 0.2008 | 3.49 | 1.372 | 1.72 | 2.297 | 0.0890 |
|  |  | 1 | 0.2979 | 0.2009 | 3.50 | 1.372 | 1.72 | 2.337 | 0.0889 |
|  |  | 2 | 0.3004 | 0.2010 | 3.50 | 1.373 | 1.72 | 2.417 | 0.0886 |
|  |  | 5 | 0.3073 | 0.2014 | 3.52 | 1.373 | 1.73 | 2.654 | 0.0881 |
| **25 °C** | *K_S* | 0.05 | 0.3399 | 0.2183 | 4.44 | 1.581 | 1.55 | 0.364 | 0.0863 |
|  |  | 0.10 | 0.3383 | 0.2181 | 4.43 | 1.580 | 1.55 | 0.366 | 0.0849 |
|  |  | 0.25 | 0.3336 | 0.2176 | 4.43 | 1.576 | 1.54 | 0.371 | 0.0826 |
|  |  | 0.50 | 0.3262 | 0.2169 | 4.41 | 1.571 | 1.54 | 0.381 | 0.0805 |
|  |  | 1 | 0.3132 | 0.2156 | 4.39 | 1.561 | 1.53 | 0.400 | 0.0784 |
|  |  | 2 | 0.2930 | 0.2140 | 4.37 | 1.549 | 1.52 | 0.434 | 0.0762 |
|  |  | 5 | 0.2564 | 0.2122 | 4.33 | 1.536 | 1.51 | 0.516 | 0.0733 |
| **20 °C** | *K_S* | 0.05 | 1.0370 | 0.0974 | 2.43 | 4.974 | 3.79 | 0.561 | 0.0434 |
|  |  | 0.10 | 1.0350 | 0.0974 | 2.43 | 4.971 | 3.79 | 0.564 | 0.0434 |
|  |  | 0.25 | 1.0290 | 0.0976 | 2.44 | 4.962 | 3.77 | 0.573 | 0.0433 |
|  |  | 0.50 | 1.0200 | 0.0978 | 2.44 | 4.946 | 3.74 | 0.587 | 0.0433 |
|  |  | 1 | 1.0050 | 0.0983 | 2.45 | 4.920 | 3.69 | 0.615 | 0.0433 |
|  |  | 2 | 0.9836 | 0.0990 | 2.46 | 4.883 | 3.62 | 0.661 | 0.0432 |
|  |  | 5 | 0.9473 | 0.0998 | 2.48 | 4.846 | 3.56 | 0.759 | 0.0427 |
| **30 °C** | *z0* | 1.00 | 0.2965 | 0.2008 | 3.49 | 1.372 | 1.72 | 2.297 | 0.0890 |
|  |  | 1.05 | 0.2965 | 0.2007 | 3.49 | 1.372 | 1.72 | 2.296 | 0.0890 |
|  |  | 1.20 | 0.2963 | 0.2006 | 3.49 | 1.371 | 1.72 | 2.292 | 0.0889 |
|  |  | 1.50 | 0.2960 | 0.2004 | 3.50 | 1.370 | 1.73 | 2.284 | 0.0888 |
|  |  | 2.00 | 0.2956 | 0.2001 | 3.51 | 1.368 | 1.73 | 2.274 | 0.0886 |
| **25 °C** | *z0* | 1.00 | 0.3262 | 0.2169 | 4.41 | 1.571 | 1.54 | 0.381 | 0.0805 |
|  |  | 1.05 | 0.3260 | 0.2167 | 4.42 | 1.569 | 1.54 | 0.381 | 0.0804 |
|  |  | 1.20 | 0.3254 | 0.2163 | 4.42 | 1.565 | 1.54 | 0.380 | 0.0802 |
|  |  | 1.50 | 0.3249 | 0.2155 | 4.42 | 1.557 | 1.54 | 0.378 | 0.0797 |
|  |  | 2.00 | 0.3250 | 0.2144 | 4.43 | 1.547 | 1.54 | 0.376 | 0.0791 |
| **20 °C** | *z0* | 1.00 | 1.0200 | 0.0978 | 2.44 | 4.946 | 3.74 | 0.587 | 0.0433 |
|  |  | 1.05 | 1.0190 | 0.0978 | 2.44 | 4.947 | 3.74 | 0.587 | 0.0433 |
|  |  | 1.20 | 1.0170 | 0.0977 | 2.44 | 4.945 | 3.75 | 0.586 | 0.0432 |
|  |  | 1.50 | 1.0130 | 0.0975 | 2.44 | 4.944 | 3.77 | 0.584 | 0.0431 |
|  |  | 2.00 | 1.0080 | 0.0972 | 2.45 | 4.942 | 3.79 | 0.582 | 0.0429 |

**Table S4.** Effect of releasing z_min or z0 as free parameters instead of fixing them at the reference values used throughout this work (z_min = 1 g g⁻¹; z0 = z_min). ΔJ is the relative change in the objective function when the parameter is released; ΔAIC is the resulting change in the Akaike Information Criterion (one additional free parameter), reported only for z_min (AIC values for the z0 release are not directly comparable, as z0 is an initial condition rather than a model parameter and was allowed to vary independently of z_min in this test). ΔAIC > 0 indicates that releasing the parameter is not supported despite a lower J. Releasing z_min improves the fit only marginally and is not supported by ΔAIC at any temperature; the fitted value also varies more than two-fold across temperatures (1.24–2.68 g g⁻¹), which is not readily consistent with a fixed physiological quantity. Releasing z0 drives the estimate to the upper bound of the search range (5.0 g g⁻¹) at 30 and 20 °C, the characteristic signature of a parameter that is not constrained by the available data.

| **Parameter**  **released** | **T (°C)** | **J**  **(fixed)** | **J**  **(free)** | **ΔJ**  **(%)** | **ΔAIC** | **Fitted value**  **(g g⁻¹)** | **Note** |
| --- | --- | --- | --- | --- | --- | --- | --- |
| **z_min** | 30 °C | 0.2965 | 0.2905 | -2.0 | +1.32 | 1.238 | *–* |
|  | 25 °C | 0.3262 | 0.3006 | -7.8 | -0.70 | 1.609 | *–* |
|  | 20 °C | 1.0202 | 0.9532 | -6.6 | -1.06 | 2.678 | *–* |
| **z0** | 30 °C | 0.2965 | 0.2941 | -0.8 | – | 5.000 | *hits upper bound (5.0)* |
|  | 25 °C | 0.3262 | 0.3248 | -0.4 | – | 1.658 | *–* |
|  | 20 °C | 1.0202 | 0.9908 | -2.9 | – | 5.000 | *hits upper bound (5.0)* |

**Table S5.** Variable-specific goodness-of-fit metrics for the Droop-based model at each cultivation temperature, corresponding to the residual and observed-versus-predicted diagnostics shown in Supplementary Figure S6. RMSE is the root-mean-square error (in the units of the corresponding variable: g L⁻¹ for biomass and substrate); NRMSE (mean) is the RMSE normalised by the mean of the observed values; NRMSE (range) is the RMSE normalised by the observed range (max − min); bias is the mean signed difference between predicted and observed values (predicted − observed, so a negative bias means the model under-predicts), and relative bias expresses this as a percentage of the mean observed value. R² exceeds 0.97 for all variables and temperatures except total secreted protein at 20 °C (R² = 0.921), where NRMSE and relative bias are also highest, consistent with the lower protein titres and correspondingly lower signal-to-noise ratio obtained at this temperature. Substrate and protein are consistently, though slightly, under-predicted at all three temperatures; biomass is under-predicted at 30 °C but over-predicted at 25 and 20 °C. In all cases the relative bias remains below 7% of the mean observed value.

| **T**  **(°C)** | **Variable** | **R²** | **RMSE** | **NRMSE**  **(mean, %)** | **NRMSE**  **(range, %)** | **Bias** | **Relative**  **bias (%)** |
| --- | --- | --- | --- | --- | --- | --- | --- |
| **30 °C** | biomass | 0.992 | 0.5341 | 7.47 | 3.54 | -0.0256 | -0.357 |
|  | protein | 0.986 | 0.0056 | 8.75 | 5.03 | -1.61×10⁻⁴ | -0.250 |
|  | substrate | 0.988 | 1.777 | 11.71 | 4.61 | -0.7888 | -5.200 |
| **25 °C** | biomass | 0.989 | 0.5517 | 8.54 | 4.18 | 0.2235 | 3.461 |
|  | protein | 0.995 | 0.0037 | 5.28 | 3.01 | -1.18×10⁻⁶ | -0.002 |
|  | substrate | 0.977 | 2.338 | 13.99 | 6.11 | -0.5981 | -3.578 |
| **20 °C** | biomass | 0.985 | 0.5546 | 10.18 | 4.68 | 0.2913 | 5.347 |
|  | protein | 0.921 | 0.0459 | 18.58 | 10.73 | -0.0054 | -2.201 |
|  | substrate | 0.979 | 2.292 | 15.21 | 5.79 | -0.9915 | -6.580 |

**Figure S1.** Specific substrate uptake rate ($\rho$) and specific growth rate ($\mu$) estimated from batch culture data at T = 20, 25 and 30 °C. Both rates were obtained by differentiating cubic smoothing splines fitted to the biomass and substrate concentration profiles, respectively. Bayer *et al*. (2019) demonstrated that this approach is robust for analytical measurement errors and sampling frequency, making it particularly suitable for the relatively sparse sampling typical of batch culture experiments. Under a Monod-type formulation, $\rho$ and $\mu$ are driven by the same instantaneous substrate concentration and are therefore always proportionally coupled. However, the estimated rates reveal a dynamic decoupling between the two: particularly during the early exponential phase, when substrate concentration is high, $\rho$ reaches its maximum values while $\mu$ remains comparatively moderate and stable. This behavior suggests that substrate uptake and biomass growth respond on different timescales and are not instantaneously coupled, which a standard Monod model cannot reproduce. This observation informed the adoption of a Droop quota framework (Droop, 1968), in which an auxiliary state variable *z* mediates the dynamic relationship between substrate uptake and growth rate, allowing the two processes to evolve independently.

**Figure S2.** Progression of dissolved oxygen tension (DOT), pH and temperature for the experimental cultures: DOT progression for batch cultures at 20, 25 and 30 °C (A), pH and temperature progression for batch cultures at 20, 25 and 30 °C (B), DOT and pH progression for the continuous culture performed at 20 °C (C).

**Figure S3.** Profile likelihood of the five kinetic parameters at each cultivation temperature. Each parameter was fixed at 15 values spanning 0.3–2.5× its optimal estimate while the remaining parameters were re-optimised; the resulting chi-square, normalised to its minimum value, is plotted against the fixed parameter value (dashed vertical line: optimal estimate; horizontal line: chi2/chi2_min = 1). A narrow, parabolic, bounded profile indicates a practically identifiable parameter, whereas a flat profile indicates that the batch data do not constrain that parameter. μmax, m and qmax show well-defined, approximately parabolic minima at all three temperatures, confirming that these parameters are practically identifiable. mP shows comparatively flat profiles throughout, consistent with the larger relative uncertainty reported for this parameter in Table 1, reflecting the limited number of measurements directly informative of protein degradation. ρmax shows asymmetric profiles that flatten markedly towards higher values, most notably at 30 °C, where glucose remains well above the half-saturation constant for most of the culture and therefore provides little information on the saturating region of the uptake kinetics.

**Figure S4.** Multi-start convergence analysis of the Droop-based model. The fit was repeated 30 times per temperature from independent random initial guesses for the five kinetic parameters, and the resulting chi-square values are shown sorted from lowest to highest (log scale) to test whether the reported estimates correspond to a unique global optimum. At 30 °C and 20 °C, all restarts converged to the same parameter vector (maximum relative spread below 0.01% and 1.5%, respectively). At 25 °C, most restarts (24/30) converged to the global optimum (maximum relative spread below 0.3%), while the remaining 6 converged to distinct, higher local optima (1–39% above the global minimum), visible as points separated from the main plateau. In all cases the global optimum is well separated from any alternative solution, confirming that the parameter estimates in Table 1 are well identified.

**Figure S5.** Comparison between the proposed Droop-type model with a conventional Monod + Luedeking-Piret model, fitted independently to the same batch data at 30, 25 and 20 °C. The conventional model combines Monod growth kinetics with a constant biomass yield and standard Luedeking-Piret production kinetics (growth- and non-growth-associated), and was fitted with the same number of free parameters and the same normalised objective function used for the Droop-type model (Table 1). Circles, squares and triangles show experimental biomass, protein and substrate data (mean ± SD, n=2 biological replicates), and dashed lines show the fitted model trajectories, with the coefficient of determination (R²) indicated for each variable. Model equations and parameter definitions are shown alongside the fits. Biomass yields on substrate were first estimated directly from the batch data (0.398, 0.347 and 0.299 g biomass g⁻¹ substrate at 30, 25 and 20 °C, respectively) and used as initial guesses for the fitted yield parameter. The Droop-type model outperformed the conventional model at every temperature (ΔAIC = 50–54 in favor of the Droop-type model), with the largest discrepancy concentrated in the biomass and substrate trajectories, consistent with the conventional model's inability to capture the delayed coupling between substrate uptake and growth.

**Figure S6.** Residual and observed-versus-predicted diagnostics for the Droop-based model, by state variable (biomass, total secreted protein, substrate) and cultivation temperature. Top row: residuals (observed − predicted) plotted against time; a horizontal line at zero indicates no systematic bias. Bottom row: observed versus predicted values, with the dashed line indicating perfect agreement (1:1). Variable-specific error metrics (R², RMSE, normalised RMSE and bias) are reported in Supplementary Table S5. Residuals show no strong systematic trend with time for any variable, though protein residuals at 20 °C are noticeably more scattered than at 30 and 25 °C, consistent with the lower R² (0.921) reported for this condition.
